# Bacterial nucleomodulins: A coevolutionary adaptation to the eukaryotic command center

**DOI:** 10.1371/journal.ppat.1009184

**Published:** 2021-01-21

**Authors:** Hannah E. Hanford, Juanita Von Dwingelo, Yousef Abu Kwaik

**Affiliations:** 1 Department of Microbiology and Immunology, University of Louisville, Kentucky, United States of America; 2 Center for Predicative Medicine, College of Medicine, University of Louisville, Kentucky, United States of America; Geisel School of Medicine at Dartmouth, UNITED STATES

## Abstract

Through long-term interactions with their hosts, bacterial pathogens have evolved unique arsenals of effector proteins that interact with specific host targets and reprogram the host cell into a permissive niche for pathogen proliferation. The targeting of effector proteins into the host cell nucleus for modulation of nuclear processes is an emerging theme among bacterial pathogens. These unique pathogen effector proteins have been termed in recent years as “nucleomodulins.” The first nucleomodulins were discovered in the phytopathogens *Agrobacterium* and *Xanthomonas*, where their nucleomodulins functioned as eukaryotic transcription factors or integrated themselves into host cell DNA to promote tumor induction, respectively. Numerous nucleomodulins were recently identified in mammalian pathogens. Bacterial nucleomodulins are an emerging family of pathogen effector proteins that evolved to target specific components of the host cell command center through various mechanisms. These mechanisms include: chromatin dynamics, histone modification, DNA methylation, RNA splicing, DNA replication, cell cycle, and cell signaling pathways. Nucleomodulins may induce short- or long-term epigenetic modifications of the host cell. In this extensive review, we discuss the current knowledge of nucleomodulins from plant and mammalian pathogens. While many nucleomodulins are already identified, continued research is instrumental in understanding their mechanisms of action and the role they play during the progression of pathogenesis. The continued study of nucleomodulins will enhance our knowledge of their effects on nuclear chromatin dynamics, protein homeostasis, transcriptional landscapes, and the overall host cell epigenome.

## Author summary

Bacterial pathogens have evolved a repertoire of diverse effector proteins that are secreted or injected into the host cell cytosol, reprogramming the host cell into a more favorable environment. Many of these pathogens possess nuclear-targeted effector proteins (nucleomodulins) that modulate host cell gene expression without altering the cellular genomic sequence. By utilizing nucleomodulins obtained through evolution, pathogens can manipulate host cell gene regulation and alter host immune response to infection. Here, we provide a comprehensive review discussing a diverse array of nucleomodulins that target and modulate the host genome through interference with chromatin dynamics, histone modifications, regulation of transcription, interference of the cell cycle, and regulation of cell signaling pathways for immune response. This unique targeting of host cell gene regulation through bacterial nucleomodulins is an emerging theme and likely the tip of an iceberg regarding host–pathogen interactions at the level of the host command center.

## Introduction

Bacterial pathogens harbor a plethora of virulence factors/toxins that aid in and promote infection, replication, and persistence within host cells. A group of these virulence factors, termed effectors, are secreted or injected/translocated from bacteria into the host cell cytoplasm through various secretion pathways and systems (Sec-pathway, Tat-pathway, and type I to VII secretion systems). Upon entering the host cell cytosol, these effectors interact with specific host proteins and modulate a wide range of cellular processes and organelle functions [1]. By secreting or injecting effector proteins, pathogens can consequentially exploit host cell functions and alter host pathways [2,3]. Host pathways altered by pathogen effector proteins include, but are not limited to: interactions with lipids and cellular membranes, vesicular trafficking, cellular metabolism, autophagy, posttranslational modification, transcription, translation, and innate and cellular immune response and signaling pathways [4–10].

Studies throughout the last 3 decades illustrate how chromatin structure and dynamics are fundamental participants in cellular gene regulation and emerging as key targets for bacterial pathogens. Chromatin is a nucleoprotein complex composed of DNA wrapped around an octamer of 4 core histones proteins (H2A, H2B, H3, and H4) [2,11,12]. These DNA–histone interactions form nucleosomes with repeating subunits which, through effective compaction and shortening of each DNA polymer, contribute to the accessibility of DNA within chromatin [2,11]. By inducing fine structural alterations at the nucleosomes, access to chromatin DNA is modulated by large supramolecular complexes, such as the transcription or replication machinery [13]. The accessibility of DNA within chromatin is highly regulated by multiple processes, which include modification of DNA and core histones by various covalent modifications as well as by noncoding RNAs and the cell cycle [12]. Histone proteins exposed outside of the nucleosomes are subject to various posttranslational modifications such as: methylation, acetylation, phosphorylation, sumoylation, and ubiquitination [14]. Multiprotein complexes that regulate chromatin structure are subjected to posttranslational modifications that govern the accessibility of DNA to bind other supramolecular complexes involved in replication, transcription, and DNA repair [12,15]. In response to infection, host cells may undergo stable, long-lasting epigenomic changes and chromatin modifications that allow for cellular dedifferentiation, carcinogenesis, tolerance, and trained immunity [16]. By influencing chromatin dynamics, pathogens can alter the host cell genome and interfere with cellular processes and defense [3,17].

**Fig 1:**
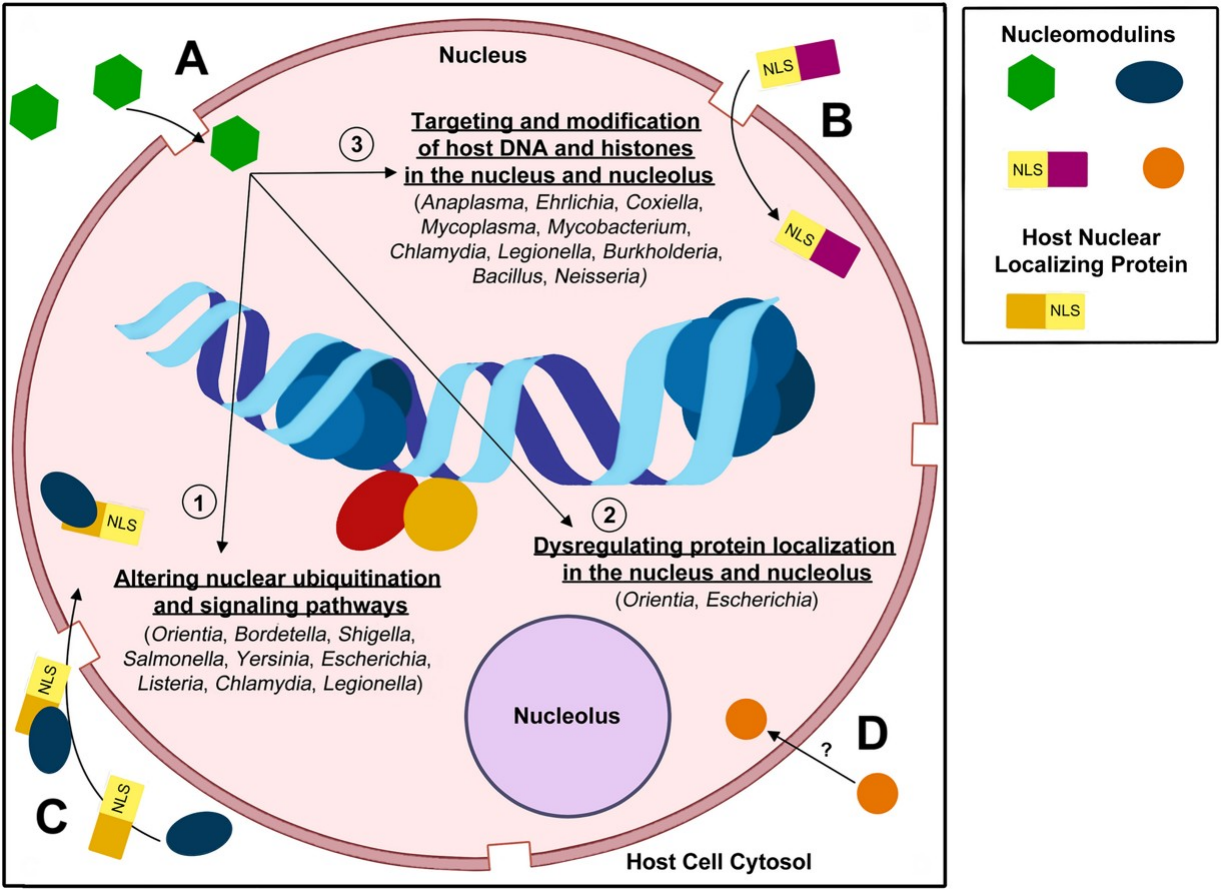
Strategies utilized by nucleomodulins to enter the nucleus and modulate host cell response and gene expression. Nucleomodulins can enter the nucleus by (A) diffusion through nuclear pores, (B) using an NLS to interact with the nuclear pore complex for import, (C) hijacking host proteins in the cytosol containing an NLS that localize to the nucleus, or (D) currently unknown mechanisms. After entry into the nucleus, nucleomodulins can modulate the host cell epigenome by (1) altering the nuclear ubiquitination and signaling pathways, (2) dysregulating protein localization and accumulation in the nucleus or nucleolus, and/or (3) directly targeting and modifying host DNA and histones. NLS, nuclear localization signal.

In the context of infection, pathogen effector proteins may be targeted towards and modulate cellular functions compartmentalized into various organelles or in the cell cytoplasm [1,3,14]. It is becoming increasingly evident that a diverse array of effector proteins induce intracellular events within organelles or the cytosol [18–20]. Through induction of these intracellular events, pathogens can modulate the host cell genome, or “epigenome,” without altering the host DNA sequence [18–20]. Effector proteins exhibiting these epigenetic changes to the host cell epigenome without altering DNA sequences have been termed by Berger and colleagues as “epigenetors” [21]. Some of the most common and well-known examples of epigenetic changes induced by epigenetors include histone acetylation and deacetylation, histone methylation, and DNA methylation [18–20,22]. While these are the most well-known instances of epigenetic modifications, it is important to note that not all epigenetic changes induced by pathogens fall under these examples [18–20,22]. Studies have defined the previously listed examples as direct strategies for inducing epigenetic modifications via altering host chromatin. However, pathogens are also capable of indirectly modulating the host cell epigenome and gene expression via targeting of host cell signaling pathways, illustrating sophisticated cross-talk between host cell epigenetic and signaling events (Fig 1) [19].

Recently, studies reveal that various bacterial pathogens have evolved numerous effectors allowing them to target host cell nuclei and modulate host epigenetic regulators. As a result, these pathogens can alter host cell transcription, translation, and overall cellular gene regulation and immune response by acting directly within the nucleus [2]. With this, there is an emerging theme that numerous bacterial effectors injected or secreted into the host cell cytosol are nuclear-targeted and have been designated by Bierne and colleagues as “nucleomodulins” [2]. These nucleomodulins function within the host cell nucleus to modulate various nuclear processes and consequently influence the host cell epigenome [2]. For this activity, nucleomodulins may directly bind host chromatin or indirectly modify chromatin structure and transcription via mimicry of transcription factors, chromatin regulatory factors, or gene expression regulators [2,3,23].

Eukaryotic proteins that translocate to the nucleus harbor a peptide motif, termed a eukaryotic nuclear localization signal (NLS), which mediates the transport of proteins into the nucleus through nuclear pore complexes [24,25]. Interestingly, several but not all bacterial nucleomodulins harbor NLS sequences that direct transport to the host nucleus [1,2]. The functional NLS is located on either the N or C terminal of a nucleomodulin and interacts with nuclear importins to enter the nucleus [26]. Terminal NLS may vary in terms of their length and features, but nearly all have short stretches of basic amino acids with the consensus sequence K-K/R-X-K/R [25]. Multiple NLS classes have been identified throughout the years, indicating there is flexibility in these signals [24]. While NLSs mediate the transport of eukaryotic proteins into the nucleus, multiple nucleomodulins are reported to enter the nucleus without a predicted NLS on either terminus (Fig 1) [2,27]. Because of this, the mechanisms used by a large number of nucleomodulins for nuclear trafficking and entry still remain unclear [2].

In this review, we discuss the currently identified nucleomodulins and explore their diverse mechanisms for nuclear-mediated modulation within eukaryotic cells (Fig 1). By doing so, we aim to share the exciting and sophisticated strategies that have evolved in different bacterial pathogens to promote survival and consequently take advantage of the host cell epigenome. Here, we will also provide speculation on the shared function and potential evolutionary convergence shared between different nucleomodulins and eukaryotic factors.

### The discovery of bacterial nucleomodulins

The first nucleomodulins ever discovered were identified in phytopathogens [28]. Phytopathogens in the genus *Agrobacterium* are best known by their direct mechanisms for manipulation of host cell gene expression [2,28,29]. These nucleomodulins were identified to function as transcription factors, directly interfering with host cell transcription or integrating themselves into host cell DNA to induce tumors, respectively [30,31]. Using T-DNA, a mobile segment of DNA, the species *A*. *tumefaciens* can alter genomic expression in plant host cells and promote uncontrolled cell proliferation [3,29]. For this activity to occur, the type IV secretion system (T4SS) of *A*. *tumefaciens* injects T-DNA and associated Vir proteins into the host cell cytosol [2]. Once in the cell cytosol, Vir proteins coating the T-DNA interact with host cell factors to promote nuclear localization of T-DNA. After localizing to the nucleus, the T-DNA of *A*. *tumefaciens* is incorporated into the plant cell genome through the induction of double-strand breaks and nonhomologous end-joining repair [2,28,29].

Virulence protein D2 (VirD2) serves as a chaperone for intracellular T-DNA transport and facilitates host genomic transformation. VirD2 contains an N-terminal and C-terminal NLS and is attached to the 5′ end of T-DNA to promote T-DNA release from the bacterium, [32,33]. VirE2 proteins comprise majority of the protective T-DNA protein coating, play an important role in packaging T-DNA into a nucleoprotein transfer complex for transport, and interact with the host plant transcription factor VirE2 interacting protein 1 (VIP1) [2,34]. While the presence of NLSs has been identified on VirE2, their roles in nuclear import have yet to be fully established [32]. Host VIP1 is suggested to be exploited during *A*. *tumefaciens* infection for its ability to interact with the nuclear import machinery for enhanced entry of T-DNA into the nucleus. Host VIP1 also interacts with core histones to mediate T-DNA targeting of host chromatin, and interactions with other bacterial proteins such as VirF, F-box, and VirE3 (a VIP1 mimic) to promote uncoating of T-DNA with host proteasomal degradation machinery [2,34]. *Agrobacterium* nucleomodulins are described to mimic host factors and function as transforming factors [32,33]. While *Agrobacterium* is the source of the first reported nucleomodulins, little is known regarding the mechanisms used by *Agrobacterium* nucleomodulins during pathogenesis and the full extent of their long-term epigenetic modulation within the host cell.

### Activation of host transcription by nucleomodulins of phytopathogens

Since the discovery of the first nucleomodulins *in Agrobacterium*, other phytopathogen species were observed to control host genes through the use of transcription activator-like effector nucleases (TALENs) [28,29,35]. *Xanthomonas* and *Ralstonia* phytopathogens inject TALENs into host plant cells via a type III secretion system (T3SS). Within the cell cytosol, the TALENs translocate to the nucleus where they bind TAL-specific DNA sequences in the host genome and induce specific host gene expression by mimicking host transcription activators [35,36]. While the contribution of TALENs to *Xanthomonas* pathogenesis has been largely characterized, their functional role during *Ralstonia* pathogenesis has yet to be explored [37].

The first nucleomodulin identified with specificity for binding directly to a eukaryotic promotor element, and the founder of the TALEN family, is AvrBs3 of *Xanthomonas* [35,37]. AvrBs3 was first discovered during the study of *Xanthomonas campestris* infection in pepper plant cells [37]. After AvrBs3’s initial discovery, an AvrBs3-like effector protein, named PthA, was later identified to elicit a similar disease phenotype as AvrBs3 from *Xanthomonas axonopodis* and gall-forming *Pantoea agglomerans* [38]. Together, AvrBs3 and PthA formed the AverBs3/PthA family of effectors distributed between *Xanthomonas* and *Ralstonia* spp. that was later renamed to “TALENs” [3,33,36].

When TALENs are injected into host cells, their translocation to the cell nuclei is mediated by 2 or 3 functional NLSs located in their C-terminal regions [33]. During *X*. *campestris* infection, AvrBs3 directly targets a conserved *UPA* box of the *upa20* promoter region within the host genome, resulting in an increase of *upregulated by AvrBs3* (*upa*) gene expression [35–37]. Within the eukaryotic genome, *upa20* encodes a transcription factor and functions as a master regulator of cell enlargement upon activation and up-regulation of *upa7*, an α-expansin-encoding gene [35–37,39]. Research findings indicate that AvrBs3 activity induces a transcriptional cascade within the host cell nucleus, reprogramming host cell development and facilitating pathogen replication and dispersal [37].

TALENs were previously proposed to be the sole effector family capable of activating plant host transcription pathways [39,40]. However, while *P*. *agglomerans* is reported to inject AvrBs3-like PthA into host cells for mimicry and modulation of host transcription factors, *P*. *agglomerans* also injects a second type of T3SS-injected effector family constituted of HsvG and HsvB with similar function [39,40]. Like TALENs, HsvG and HsvB are suggested to function as potential transcription factors within plant host cells [39,40]. HsvG and HsvB are paralogous effectors found in *P*. *agglomerans* pv. *gypsophilae* and *P*. *agglomerans* pv. *betae*, respectively [40]. Both effectors contain 2 NLSs at their N- and C-terminal regions for translocation into host nuclei. They are distinguished from each other by the presence of 2 direct repeat sequences of amino acids in the transcription activation domain of HsvG compared to the single direct repeat in the transcription activation domain of HsvB [40]. HsvG and HsvB are described as DNA-binding proteins that have been previously observed to activate transcription within yeast and are hypothesized to do the same within plant host cells [33,40]. Recent studies performed with *P*. *agglomerans* infection of gysophila and beet cells have identified potential transcription activator-like activity of HsvG and HsvB, respectively, after translocation to host cell nuclei [33,40]. With this potential identification, research has focused on determining how HsvG alters transcription within gysophila cells.

Upon injection of HsvG into gysophila cells, HsvG was found to target genes within the DnaJ protein family, termed “HSVGT” [40]. The DnaJ family of proteins is known to be widely distributed among both prokaryotes and eukaryotes [40]. DnaJ proteins typically possess a J-domain responsible for performing chaperone activity and act as co-chaperones for the heat-shock protein, Hsp70, involved in the cellular chaperone network and cellular stress response [40]. However, unlike most proteins within the DnaJ family, HSVGT lacks the characteristic J-domain; therefore, HSVGT is not expected to be involved in activation of Hsp70 [40]. Instead, HSVGT is hypothesized to serve as a transcription factor within plant cells [40]. HsvG reportedly binds to the *HSVGT* promotor of the gysophila genome and, as a result, represses transcription of defense-associated plant genes to promote *P*. *agglomerans* infection [39]. The capacity of HsvG to bind HSVGT, and the induction of further *HSVGT* expression after HsvG translocation to the plant nucleus, supports the hypothesis that HsvG of *P*. *agglomerans* acts as a host transcriptional factor during infection [37]. While the target of HsvG for host transcription activation has been identified, further studies are necessary to better characterize the mechanism used by *P*. *agglomerans* to manipulate host cell gene expression.

Altogether, this information evaluates how both TALENs and HsvG nucleomodulins evolved in phytopathogens and consequently alter host transcription activation. By harboring nucleomodulins capable of mimicking and modulating host transcription factors, *Xanthomonas*, *Ralstonia*, and *Pantoea* spp. may induce short- or long-term epigenetic effects on the host cell epigenome that promote pathogenesis. A more comprehensive list of nucleomodulins identified in phytopathogens and their functions is summarized in Table 1.

**Table 1:** Phytopathogen effectors that target the nucleus.

Pathogen	Effector	Effector Function	Source
*Agrobacterium tumefaciens*	VirD2	Binds to and chaperones bacterial T-DNA into host cell nucleus	[41,42]
	VirE2	Interacts with host VIP1 transcription factor to promote T-DNA import into the nucleus. Potentially acts as a plant transcriptional activator through interacting with the plant-specific transcription factor (pBrp)	[27,34]
	VirE3	Mimics host VIP1, facilitates nuclear import of VirE2, and interacts with host pBrp. Suggested to modulate plant gene activation	[27,43]
	VirF	Mimics the substrate recognition subunit of the SCF and hijacks the SCF to strip the T complex of its escort proteins. Functions as an F-box protein and host transformation factor	[44,45]
	Protein 6b	Reportedly targets the nuclear proteins NtSIP1, NtSIP2, histone H3, SERRATE, and AGO1. Disrupts the host cell microRNA pathway cells and interferes with gene expression	[2,46]
*Pantoea agglomerans*	HsvB	DNA-binding protein; likely acts as a transcription factor	[39,47]
	HsvG	Binds to the *HSVGT* promotor of host DNA and represses transcription of defense-associated plant genes; likely acts as a transcription factor	[39,40,47]
	PthA	AvrBs3-like effector; mimics host transcription factors to modulate cell development	[38]
*Phytoplasma*	Sap11	Binds and destabilizes TCP 1 and 2 transcription factors of Arabidopsis. These control plant development and promote expression lipoxygenase (LOX) genes which are involved in jasmonate (JA) synthesis	[48]
*Pseudomonas syringae*	HopAI1	Represses host defense signaling by deactivating MAPK	[49]
	HopBB1	Interacts with transcription factor TCP14. Uses this interaction to target TCP14 to the SCF^COI1^ degradation complex by connecting it to JAZ, a JA signaling repressor	[50]
	HopQ1	Contains nucleoside hydrolase-like domain; Induces cell death in certain hosts and enhanced disease in others through unknown mechanisms	[51,52]
*Ralstonia solanacearum*	Brg11	Transcription activator-like effector	[53]
	PopP2	Alteration of host gene transcription through acetylation; Regulation of host cell defense machinery through (de)acetylation	[54]
	RipAB	Suppresses Ca2+ signaling pathway at the transcriptional level to promote infection	[55]
*Xanthomonas*	AvrBs3	Targets the master regulator of cell enlargement, *upa20*; induces transcriptional cascade and modulates host cell development	[35,37]
	AvrHah1	Transcription activator-like effector; triggers Bs3-dependent hypersensitive response	[56]
	AvrXa5	Transcription activator-like effector	[38]
	AvrXa7	Transcription activator-like effector that activates members of the SWEET sucrose uniporters through recognition of effector-binding elements located in promoter regions	[57]
	AvrXa10	Transcription activator-like effector	[38,58]
	PthXo1	Transcription activator-like effector; acts as a transcription factor and induces expression of SWEET11	[59]
	XopD	Alters host gene transcription through binding and modifies chromatin structure; expression in nucleus results in redistribution of nuclear proteins	[60,61]

JA, jasmonate; LOX, lipoxygenase; MAPK, mitogen-activated protein kinase; SCF, SKP1-CULLIN1-F-box; VIP1, VirE2 interacting protein 1; VirD2, Virulence protein D2.

### Alteration of host nuclear homeostasis by nucleomodulins

Besides phytopathogens, the number of nucleomodulins identified in mammalian pathogens has been increasing throughout the years. Mammalian bacterial pathogens have recently been identified to target and modulate eukaryotic nuclear ubiquitination and signaling pathways necessary for regulating nuclear protein homeostasis [62,63]. *Orientia tsutsugamushi*, *Bordetella pertussis*, *Shigella flexneri*, *Salmonella enterica*, and *Escherichia coli* are all pathogens found to secrete effector proteins that localize to host cell nuclei and alter cellular homeostasis by targeting ubiquitination. *Yersinia* and other nucleomodulins of *E*. *coli*, on the other hand, modulate nuclear protein homeostasis through other methods such as targeting nuclear signaling pathways and ribosomal biogenesis. *O*. *tsutsugamushi* (previously *Rickettsia tsutsugamushi*) [64,65] secretes one of the largest repertoires of ankyrin (Ank) protein paralogs through a type I secretion system (T1SS). Of this repertoire, 2 Ank groups are reported to be involved in modulating host cell ubiquitination pathways in the nucleus [3,66–68]. Ank1 (specifically the 1A, 1B, 1E, 1F, 1U4, 1U5, and 1U9 paralogs) and Ank6 are 2 groups of *O*. *tsutsugamushi* Ank proteins found to localize in the nuclei of HeLa cells and primary macrophages [3,66–68]. While these potential nucleomodulins are found within host cell nuclei, the mechanisms behind their nuclear translocation have yet to be determined. Nearly all Ank proteins of *O*. *tsutsugamushi* harbor N-terminal Ank repeats and a C-terminal F-box domain addressed as the “pox protein repeats of ankyrin C terminus” (PRANC) motif. This domain interacts with CULLIN-1 and SKP1 core components of the SKP1-CULLIN1-F-box protein (SCF) E3 ubiquitin ligase complex [3,62,66,67]. The Ank domain of Ank1 is suggested to bind cell-specific target substrates in the nucleus, while the F-box binds and recruits SKP1 to promote substrate degradation [3,66–68]. By interacting with host ubiquitin ligase complexes, the multiple paralogs of Ank1 localizing to the nucleus are suggested to modulate diverse host cell functions during *O*. *tsutsugamushi* infection [67].

More recently, Ank1 and Ank6 of *O*. *tsutsugamushi* and the T3SS-injected BopN effector of *B*. *pertussis* are described to modulate host cell NF-κB by inhibiting nuclear accumulation of the p65 subunit of NF-κB [68,69]. Ank1 and Ank6 translocate to host cell nuclei via the nuclear importin β1 pathway [3,68]. The method used by BopN for nuclear translocation is still unknown. The domains of Ank1, Ank6, and BopN responsible for nuclear import have yet to be identified. Upon entering the nucleus, Ank1, Ank6, and BopN are suggested to directly bind and inhibit nuclear p65 or promote the nuclear export of p65 through interactions with exportin 1 [68,69]. By inhibiting nuclear accumulation of p65, these nucleomodulins can manipulate the NF-κB pathway and repress the antimicrobial response of host cells [68]. While the specific molecular mechanisms used by Ank1, Ank6, and BopN have yet to be thoroughly characterized, these effectors are hypothesized to interact with ubiquitin ligase complexes and antagonize or mediate ubiquitination of host cell NF-κB. BopN is also suggested to promote nuclear translocation of the p50 subunit of NF-κB for up-regulation of host IL-10 production and alter MAPKs, allowing BopN to regulate various host cell transcription factors [69]. By sharing similar function as Ank1/Ank6 and altering host immune response, BopN activity promotes an immunosuppressive host environment and facilitates the colonization and proliferation of *B*. *pertussis* [69]. While more research is necessary to characterize the molecular mechanisms utilized by Ank1, Ank6, and BopN for regulation of host NF-κB pathways, the current understanding of these nucleomodulins suggests the potential of pathogens sharing epigenetic-altering effector function obtained through convergent evolution or horizontal gene transfer.

*S*. *flexneri* secretes effectors proteins from a T3SS to promote invasion of intestinal epithelial cells. Of the 5 nucleomodulins reported to be secreted from *S*. *flexneri*, two are involved in altering nuclear ubiquitination. IpaH_9.8_ is the only effector of the *Shigella* IpaH effector group described to be a nucleomodulin. While IpaH_9.8_ lacks a predicted NLS, studies of intestinal epithelial cells have found that IpaH_9.8_ nuclear localization is dependent on host cell microtubules [70–72]. However, the molecular mechanism behind microtubule-mediated import of IpaH_9.8_ into the nucleus has yet to be described. IpaH_9.8_ contains leucine-rich repeat (LRR) motif, also described as the LPX-domain, present on its N-terminus responsible for recognizing cell substrates and acting as a protein-binding domain [66,73,74]. As a member of IpaH *shigella* effectors, IpaH_9.8_ possesses a C-terminal E3 ubiquitin ligase domain (NEL domain) necessary for ubiquitination of the nuclear U2AF mRNA splicing factor and inhibits U2AF-dependent splicing reactions [3,71–73]. As a result of binding and ubiquitinating U2AF, IpaH_9.8_ negatively regulates the expression of genes involved in proinflammatory response and neutrophil recruitment to the site of infection [73]. Genes repressed by U2AF ubiquitination include, but are not limited to, *il-8*, *RRANTES*, *GM-CSF*, and *il-1β* [71,73,74].

IpaB is a second nucleomodulin of *Shigella* involved in altering nuclear ubiquitination. Unlike the 4 other *Shigella* nucleomodulins, IpaB is unique in its ability to function as a cyclomodulin that alters the host cell cycle. While IpaB localizes to the nuclei of infected HeLa and intestinal epithelial cells, IpaB lacks a predicted NLS [75,76]. Because of this, IpaB is hypothesized to rely on complex formation with Mad2L2 for nuclear import [75,77,78]. Mad2L2 is an NLS-containing inhibitor of the anaphase-promoting complex/cyclosome (APC) ubiquitin ligase complex responsible for aiding in cell cycle progression [3,75,77,78]. Interaction of IpaB with Mad2L2 in the nucleus promotes activation of APC^Cdh1^, results in a premature degradation of APC^Cdh1^ substrates, and delays mitotic progression [75]. This IpaB/Mad2L2-mediated arrest is suggested to promote *Shigella* colonization of intestinal epithelial cells [78]. By interacting with Mad2L2, IpaB modulates host cell ubiquitin complex activity and, as a result, promotes *Shigella* colonization of normally rapid-proliferating epithelial cells.

SspH1 of *S*. *enterica* serovar Typhimurium is another nucleomodulin, like IpaH_9.8_ from *Shigella*, identified to contain an LPX and NEL domain. During infection, SspH1 is translocated into the host cell by both the SPI1 and SPI2 T3SS of *S*. *enterica* [79–83]. After translocation into the host cell cytosol, SspH1 is then trafficked to the host cell nucleus [84–87]. Because SspH1 lacks a classical NLS, nuclear import of SspH1 is suggested to be mediated by interactions with human serine/threonine protein kinase 1 (PKN1), responsible for phosphorylation of TNF receptor-associated factor 1 (TRAF1) when activated [85,86]. By phosphorylating TRAF1, PKN1 suppresses TRAF1 function in IKK/NF-κB and JNK signaling and inhibits the NF-κB signaling pathway [86,87]. The specific molecular interactions used by SspH1 to inhibit the NF-κB signaling pathway through PKN1 activation has yet to be thoroughly characterized, but the NEL domain of SspH1 is found necessary for this inhibition to occur [85]. Due to SspH1’s similarities with IpaH_9.8_ and description as an IpaH_9.8_ orthologue, SspH1 is suggested to function as an ubiquitin ligase responsible for targeting and ubiquitinating PKN1 [2,3,84,86]. By modulating the NF-κB pathway through PKN1 activation, SspH1 represses NF-κB-dependent gene expression and inhibits the host inflammatory response to promote *S*. *enterica* pathogenesis.

Members of the T3SS-injected NleG effector family of EPEC and enterohemorrhagic (EHEC) *E*. *coli* share functional similarity to IpaH effectors from *Shigella* [88,89]. The NleG effector family comprises majority of the core effector repertoire in *E*. *coli* and is the largest family of effectors injected by EHEC [90–94]. Recently described as a family of U-Box E3 ubiquitin ligases, NleG effectors target distinct host proteins for degradation and provide versatile scaffolding for host–pathogen interactions [90,95]. *E*. *coli* NleG effectors possess a unique N-terminal domain necessary for substrate interactions and has yet to be found in any other E3 ubiquitin ligases [91]. The C-terminal domain of NleG effectors is reportedly analogous to eukaryotic RING/U-box domains and necessary for NleG E3 ligase activity [96]. NleG5-1 is an effector of the NleG family recently identified as a nucleomodulin [91]. While NleG5-1 lacks a predicted NLS, it is smaller than 60 kDa in size and hypothesized to enter host cell nuclei through nuclear pore diffusion [91]. In the nucleus, NleG5-1 targets the Mediator complex member MED15, responsible for serving as an end point where various cell signaling pathways for RNA polymerase II-dependent transcription converge [3,91]. TGF-β and SREBP1 are two of the transcription signaling pathways affected by NleG5-1 targeting of Med15 [3,91]. By disrupting TGF-β and SREBP1 signaling pathways, NleG5-1 promotes disruption of epithelial cell tight junctions and intracellular lipid homeostasis, respectively [91]. By performing ubiquitin-mediated degradation of Med15, NleG5-1 is yet another E3 ubiquitin ligase nucleomodulin representing a multifunctional mechanism shared by various pathogens for modulating host epigenetics and promoting a favorable niche for pathogenesis.

EPEC and EHEC are also recently reported to inject a novel T3SS effector protein called cyclin-inhibiting factor (Cif). Cif is a modular protein composed of an exchangeable N-terminal secretion and translocation signal. During *E*. *coli* infection of epithelial cells, Cif is described to localize within host cell nuclei where, like IpaB, it functions as a cyclomodulin [2,3,7,97]. After localizing within the nucleus, Cif demonstrates deaminase enzymatic activity targeted towards the host ubiquitin-like protein, NEDD8 [7,98]. NEDD8 deamination, mediated by Cif, impairs NEDD8 conjugation with CULLIN and results in an inhibition of NEDD8-modified CULLIN-RING ubiquitin ligase (CRL) activity [3,7,98]. As a consequence of Cif-mediated inhibition of CRL activity, the host cell fails to ubiquitinate proteins p21 and p27 for ubiquitin-dependent degradation by the 26S proteasome, an event necessary for cell cycle progression [2,7,98]. Injection of Cif and induction of cell cycle arrest delays gut epithelial cell turnover and promotes *E*. *coli* colonization [2,7,98]. Cif homologues presenting similar function were recently identified in *Yersinia pseudotuberculosis*, *Photorhabdus luminescens*, *Photorhabdus asymbiotica*, and *Burkholderia pseudomallei* [7]. The Cif and IpaB nucleomodulins demonstrate a unique evolutionary convergence between various bacterial pathogens used to modulate host cell homeostasis through epigenetic strategies. Because these pathogens utilize a T3SS for nucleomodulin injection, it is interesting to speculate how a shared epigenetic strategy for infection persistence came to be.

EspF is a third T3SS-injected effector protein translocated from EPEC into host cell nuclei and is the first bacterial effector recognized to target the nucleolus [99]. EspF is previously described as a mitochondrial-targeted effector protein involved in the disruption of intestinal epithelial cell junctions, inducing cell injury, and promoting apoptosis during EPEC infection [100,101]. During early stages of infection, EspF accumulates in the host cell mitochondria in a functional mitochondrial membrane potential (MMP)-dependent manner and results in a loss of MMP [99–102]. However, during late stages of infection, EspF is reported to traffic to the nucleolus [102]. Nucleolar translocation of EspF is dependent on the N-terminal NLS, specifically located at residues 21 to 41 [102]. How EspF translocates from the host cell mitochondria to the nucleolus is still unclear. After localization to the nucleolus of HeLa cells, EspF activity induces a significant redistribution of an abundant nonribosomal protein, nucleolin [102]. Nucleolin is redistributed from the cellular compartments of transfected and infected cell cultures into the cytoplasm [102]. In correlation with EspF-induced loss of nucleolin from the nucleolus, HeLa cells exhibit an altered distribution of small nuclear RNA U8 and inhibition of pre-RNA processing [99,102]. Overall, EspF translocation to the nucleolus of HeLa cells results in a shutdown of host ribosome biogenesis and increased access of nutritional resources for EPEC. As the first bacterial effector identified to target the nucleolus, EspF illustrates a novel mechanism evolved in EPEC that consequently alters nuclear protein homeostasis and promotes pathogen growth and intracellular persistence.

YopM of *Yersinia* (*Y*. *pestis*, *Y*. *pseudotuberculosis*, and *Y*. *enterocolitica*) is a third nucleomodulin that possesses an LPX domain similar to that found in IpaH_9.8_ and SspH1 [103]. After injection via a T3SS, YopM localizes to host cell nuclei through a vesicular-associated pathway [104,105]. YopM from *Y*. *enterocolitica* is described to contain 2 putative NLSs within its sequence and rely on DDX3/CRM1 interactions for nuclear export [104,105]. YopM is proposed to function as an E3 ubiquitin ligase in 1 strain of *Y*. *pestis* [3,106]. However, due to the lack of an NEL domain, YopM is suggested to primarily serve as a scaffolding protein that interacts and forms a complex with ribosomal S6 protein kinase 1 (RSK1) and protein kinase C-like 2 (PRK2) within host cell cytosol and nuclei [107–110]. YopM recruits RSK1 and PRK2 into the YopM-RSK1-PRK2 complex through binding interactions with its C-terminal amino acids and LPX domain, respectively [105,108,111,112]. Upon complex formation, YopM induces hyperphosphorylation of the YopM-RSK1-PRK2 complex and reportedly protects RSK1 from dephosphorylation [107,110]. Hyperphosphorylation of RSK1 via YopM in the nucleus is associated with an increased expression of immunosuppressive cytokine genes including *IL-10* [105]. As for PRK2 activation, it is suggested that cytosolic PRK2 in complex with YopM and RSK1 is phosphorylated by RSK1 [107]. Once activated, PRK1 and RSK1 activities induce phosphorylation of the cytosolic substrate pyrin, which serves as an important regulator for inflammasome formation [113–115]. By inducing downstream phosphorylation of pyrin, YopM inhibits pyrin inflammasome formation and promotes *Yersinia* evasion of the host inflammatory response [113–116]. Another function that is partially elucidated for YopM is that it suppresses the transcription of the proinflammatory cytokine TNFα and is associated with decreased production of IFN-γ and increased levels of IL-18 [104,108]. However, more research is required to fully understand the molecular mechanisms behind this function. Overall, YopM serves as a multifunctional nucleomodulin crucial for dampening the host inflammatory response and promoting *Yersinia* virulence within the host. While the functions of YopM vary based on its subcellular localization, host protein target, and infected cell type, it remains to be explored how these complex features of YopM contribute to the pathogenicity of *Yersinia*, and to what extent YopM alters host gene expression, throughout the course of infection.

Recently, a small group of zinc metalloproteases were identified in *S*. *enterica* serovar Typhimurium and *E*. *coli* (EPEC and EHEC) that function to preserve host cell homeostasis by targeting nuclear signaling pathways. GtgA, GogA, and PipA from *S*. *enterica* and NleC from *E*. *coli* are T3SS-injected nucleomodulins that reportedly contain short metal-binding motifs for active-site zinc [66,117]. While these metalloproteases were identified to localize to the nuclei of transfected and infected cells, there is little known regarding the mechanisms used for nuclear translocation [118,119]. Studies have shown that PipA, GtgA, GogA, and NleC redundantly target components involved in the NF-κB signaling pathway [117,119,120]. PipA, GogA, and GtgA cleave RelA (p65), RelB, and occasionally cRel nuclear transcription factors, but do not cleave NF-κB1 (p105/p50) or NF-κB2 (p100/p52) [117]. NleC, on the other hand, reportedly cleaves all 5 NF-κB subunits and is the first bacterial effector described to facilitate cleavage and degradation of histone acetyltransferase p300 [3,18,121]. Without an accumulation of p300 in the nucleus, NleC contributes to the suppression of IL-8 secretion of host cells [121]. As a result of targeting substrates of the NF-κB pathway, PipA, GogA, GtgA, and NleC promote regulation of the host cell transcriptional response and inhibit IL-8 gene expression. While these metalloproteases from *S*. *enterica* and *E*. *coli* share some degree of substrate specificity, they present few similarities in sequence identity. Due to sharing low sequence identity, *S*. *enterica* and *E*. *coli* are speculated to have separately evolved similar strategies for regulating host nuclear homeostasis to promote pathogenesis [117]. Further studies are required to fully comprehend the advantage of nucleomodulin-mediated regulation of host cell homeostasis and how host substrate recognition is evolutionarily shared among several *S*. *enterica* and *E*. *coli* effectors for epigenetic regulation within host cells.

### Association of nucleomodulins with nuclear proteins and modulation of host responses

During infection, several nucleomodulins from *Listeria monocytogenes*, *S*. *flexneri*, *Chlamydia psittaci*, and *Legionella pneumophila* associate with nuclear proteins and indirectly impact host chromatin structure and regulatory processes. *Listeria* nuclear targeted protein A (LntA) is the first nucleomodulin identified in *L*. *monocytogenes* [3,23,122]. LntA contains a central NLS and induces host epigenetic modifications by interacting with components of chromatin-associated complexes [122,123]. Research to identify a binding partner for LntA led to the characterization of a novel chromatin repressor, bromo adjacent homology domain-containing 1 (BAHD1) protein. BAHD1 serves as a scaffolding protein and complexes with heterochromatin proteins, histone methyltransferases, and histone deacetylases (HDACs) to condense chromatin into heterochromatin and silence gene expression [3,23,122]. While the genes silenced by BAHD1 activity vary depending on cell type and cell signaling, BAHD1 is identified to repress the expression of interferon stimulated genes (ISGs) in epithelial cells [3,123]. To do so, BAHD1 complexed with HADC is recruited to ISGs and induces histone H3-targeted deacetylation and transcriptional repression [23]. When epithelial cells are infected with *L*. *monocytogenes*, LntA binds to a proline rich region of BAHD1 via an elbow domain in its 5-helix bundle structure and inhibits complex formation with HADC [123]. By inhibiting BAHD1-complex formation, BAHD1 is unable to bind ISG promotors or deacetylate histone H3 for gene silencing [1]. Thus, LntA of *L*. *monocytogenes* prevents BAHD1-mediated gene silencing, promotes H3 acetylation, and stimulates the expression of ISGs for modulation of the host cell immune response [23,66,124].

After LntA, OrfX is the second nucleomodulin to be discovered in *L*. *monocytogenes* [3,23,66,124]. Like many other nucleomodulins, OrfX lacks a predicted NLS, and its mechanism for entering the nucleus has yet to be identified. However, once inside the nucleus, OrfX reportedly targets the Ring1 YY1-binding protein (RYBP) [66,124]. RYBP is a conserved, multifunctional zinc finger protein responsible for regulating gene expression at the transcriptional level and is an essential regulator for vertebral embryonic development [23,124]. Overall, RYBP’s functions include, but are not limited to: (1) mediating protein–protein interactions in epigenetic complexes such as the BCL6 corepressor (BCOR) complex and (2) mediating interactions between YY1 and E2F transcription factors for activation of the Cdc6 promotor involved in DNA replication [3,23,66,124]. RYBP also functions by preventing proteasomal degradation of the p53 tumor suppressor via binding with ubiquitin E3 ligase MDM2 and promotes cell apoptosis through interactions with cell and viral proteins (procaspase-8, procaspase-10, Fas-associated death domain (FADD), fibronectin type III and ankyrin repeat domains 1 (FANK1), and viral apoptin) [3,23,66,124]. It is important to note that the p53-MDM2 pathway modulates intracellular levels of reactive nitrogen species (RNS) and reactive oxygen species (ROS) for immune defense within macrophages [3,23,66]. With this in mind, it is proposed that OrfX indirectly dampens the oxidative activity of macrophages in vitro and, potentially, other cell functions through targeting RYBP [66,124]. Because increased OrfX expression in macrophages correlates with reduced levels of RYBP in infected macrophages, it is hypothesized that OrfX targets RYBP for degradation and, as a result, inhibits p53 activity and other downstream functions of RYBP to promote intracellular pathogen survival [66]. The specific mechanism of action used by OrfX to interact with RYBP has yet to be elucidated. Together, LntA and OrfX modulate host gene expression by indirectly targeting host chromatin and regulating the activity of host nuclear proteins, promoting *Listeria* pathogenesis.

Of the many effector proteins injected by *S*. *flexneri*, 5 different effectors are currently proposed as nucleomodulins and are critical in promoting pathogenesis [3]. While one of these effectors, OspC1, has been identified to localize to the nucleus and influence epithelial and HeLa cell signaling for polymorphonuclear neutrophil (PMN) migration, there has yet to be conclusive evidence suggesting OspC1’s specific mechanism of action within the nucleus [70,125,126]. Two other *Shigella* nucleomodulins reported to target and modify important nuclear proteins are OspF and OspB [3,70,126]. OspF is a nucleomodulin that down-regulates the host innate immune response during *S*. *flexneri* infection [125]. No obvious NLS has been detected for OspF. However, OpsF is hypothesized to possess a novel nuclear transit peptide in its C-terminal domain that serves as a nonclassical NLS [125]. This transit peptide was found to bind nuclear importin α1 in HeLa cells and promote nuclear transportation of OspF in an importin α1-dependent manner [125]. The role of this transit peptide during nuclear localization of OspF in epithelial cells has yet to be explored.

After entering the nucleus of epithelial cells, OspF targets and irreversibly dephosphorylates mitogen-activated protein kinases (MAPKs) through its phosphothreonine lyase activity [127]. OspF inhibits host MAPK signaling through beta elimination of a phosphate group and converts the MAPK phosphothreonine residue to dehydrobutyrine (Dhb) lacking a vital -OH group and locking MAPK in an inactive conformation [2,127,128]. By inhibiting MAPK along with ERK1/2 signaling pathways, OspF prevents downstream phosphorylation of histone H3 at a group of NF-κB-regulated promotors [2,126,127]. As a result, chromatin conformation is modified in such a way where access to NF-κB-regulated gene promotors for proinflammatory chemokines and cytokines is blocked, preventing transcription activation [1,2,124,126]. OspF inhibition of MAPK and ERK1/2 signaling pathways also prevents downstream phosphorylation of heterochromatin protein 1γ (HP1-γ), a chromatin regulator for posttranslational modifications (PTMs) [1,129]. This further disrupts active transcription of proinflammatory genes and inhibit the host’s response to infection [1,129].

The OspF and OspB effectors of *Shigella* both lack an obvious NLS domain [71]. However, it is speculated that a nuclear transit peptide on the N-terminus functions as a nonclassical NLS or interacts with host proteins to target the nucleus from the cytoplasm [71]. OspB, along with OspF, interact with nuclear human retinoblastoma protein (pRB) of epithelial cells [66,71,129]. pRB plays an important role in regulating the cell cycle, repressing gene transcription, and modulating chromatin dynamics and structure by binding with chromatin-remodeling factors [129,130]. While the molecular mechanisms of OspF and OspB interaction with pRB have yet to be identified, these 2 nucleomodulins likely function in synergy to alter chromatin structure at specific genes and down-regulate host innate immunity [66,71,129].

Secreted inner nuclear membrane-associated *Chlamydia* protein (SinC) is a novel nucleomodulin injected by *C*. *psittaci* into host cells through a T3SS [131,132]. Unlike the currently known *Chlamydia* effector proteins, SinC possesses 2 unique properties. First, SinC is found to localize to the inner nuclear membrane during the late stages of infection where it interacts with the nucleoporin ELYS, lamin B1, LEM (LAP2, emerin, MAN1) domain proteins, lamin-associated polypeptide 1 (LAM1), and lamin B receptor (LBR) [133,134]. Due to SinC lacking a predicted NLS or transmembrane domain, the mechanism of its nuclear translocation has yet to be determined [131]. Second, SinC undergoes intercellular transmission to neighboring cells, where it will localize to the host cell inner nuclear membrane [131,133]. During studies with HeLa and HEK293 cells, SinC was found to specifically target 4 inner nuclear membrane proteins (emerin, MAN1, LAP1, and LBR) [131]. Because emerin, MAN1, LAP1, and LBR play a major role in regulating nuclear structure and chromatin organization, it is speculated that SinC interacts with these proteins to indirectly alter chromatin structure and silence specific host response genes [131,135]. While SinC orthologues with similar function have been identified in *Chlamydia caviae* and *Chlamydia abortus*, the SinC orthologue of the well-known *Chlamydia trachomatis* does not localize to the nuclear envelope [133,136,137]. Injection of SinC into the host cell cytosol modulates gene expression in infected and neighboring host cells, promoting virulence and pathogenicity of *C*. *psittaci*. To date, the molecular interactions between SinC and host nuclear proteins during *C*. *psittaci* infection have yet to be fully assessed.

AnkH, SnpL, and AnkX are effector proteins injected by *L*. *pneumophila* through a T4SS that target host nuclear proteins and interfere with transcriptional events. While all 3 are found to localize within host cell nuclei, none of them harbor an identifiable NLS and the specific mechanism for their nuclear translocation has yet to be determined [138,139]. In the nucleus, AnkH interacts with host La related protein 7 (LARP7) through the β-hairpin loops of the third ankyrin repeat domain [140]. LARP7 is a highly conserved component of the eukaryotic 7SK small nuclear ribonucleoprotein (snRNP) transcriptional regulatory complex involved in regulating the pausing and transcriptional activity of RNA polymerase II [3,141–143]. The pause of transcriptional elongation by the 7SK snRNP complex is mediated by sequestration of the P-TEFb component, which prevents phosphorylation of RNA polymerase II to maintain a paused state [142,143]. As a result, the process of transcriptional elongation is halted at RNA polymerase II pause sites. The interaction between AnkH and LARP7 inhibits the formation of the 7SN snRNP complex [3,66,138]. This inhibition results in a prolonged pause of transcriptional elongation and an overall global reprogramming of the host transcriptional landscape [3,66,138]. Since AnkH promotes intracellular proliferation of *L*. *pneumophila* within amoeba and macrophage hosts, the AnkH-LARP7 interaction demonstrates how pathogens have evolved to target and modulate the host transcriptional response for facilitation of pathogen replication.

The SnpL nucleomodulin of *L*. *pneumophila* is another example of pathogen evolution with nucleomodulin-mediated control of RNA polymerase II activity. During *L*. *pneumophila* infection or ectopic expression, SnpL targets and directly binds host cell Suppressor of Ty5 (SUPT5H) upon localization within the nucleus [139]. SUPT5H is a component of the 5,6-Dichlorobenzimidazole 1-β-D-ribofuranoside (DRB) sensitivity-inducing (DSIF) complex and acts as a selective inhibitor in regulating the promotor proximal pausing of RNA polymerase II [3,66,139]. While SnpL activity results in an up-regulation of a variety of cell activities due to global gene activation in macrophages, the role of SnpL during *L*. *pneumophila* infection of amoeba has yet to be elucidated [139]. The SnpL-SUPT5H interaction up-regulates genes for fundamental biological processes (i.e., cell division, adhesion, survival) [139]. SnpL is hypothesized to drive mRNA expression in amoeba and influence the cell cycle phase for facilitation of pathogen replication at the cost of host cell survival [139]. However, more research is required in order to fully comprehend the biological consequences of SnpL activity on the host transcriptome in macrophages and amoeba.

AnkX is a potential nucleomodulin injected by *L*. *pneumophila* targeted towards host nuclear proteins, resulting in modulation of the host epigenome. AnkX is previously described as a phosphocholine transferase that targets and covalently modifies host cell Rab1 and Rab35, interfering with endocytic recycling and preventing fusion of the *Legionella*-containing vacuole (LCV) with host cell lysosomes [144,145]. Recently, AnkX was discovered to colocalize with host cell PLEKHN1 in the nucleus. PLEKHN1 is an endogenous cell protein found to speckle the inside of HEK293T cell nuclei [3,146]. During ectopic expression, the central region of AnkX was necessary for the targeting and binding of PLEKHN1 [146]. While little is known regarding the consequences of AnkX-PLEKHN1 interactions, PLEKHN1’s association with various proteins involved in the inflammatory response has led to the hypothesis that AnkX potentially functions as a novel nucleomodulin capable of simultaneously preventing LCV-lysosomal fusion in the cytosol and manipulating host inflammatory response from within the nucleus [146].

### Modification of host DNA by nucleomodulins

Along with utilizing interactions with nuclear proteins to indirectly modulate the host response, few nucleomodulins from *Anaplasma phagocytophilum*, *Ehrlichia* spp., *Coxiella burnetii*, *Mycoplasma hyorhinis*, and *Mycobacterium tuberculosis* may also target and directly bind to host DNA. The first Rickettsial nucleomodulin identified to directly bind host DNA and recruit histone modifying enzymes to chromatin was ankyrin repeat protein A (AnkA) [1,147,148]. AnkA is a T4SS effector protein injected by *A*. *phagocytophilum*. After injection into the host cell cytosol, AnkA translocates to the nucleus of granulocytes and directly associates with host cell DNA [1,147,148]. While AnkA is confirmed to localize within infected cell nuclei, studies have yet to identify a clear NLS [147,149]. However, studies show ANK repeats are capable of functioning like an NLS [147]. In support of this assessment, transfection of HEK293T cells with modified AnkA revealed the N-terminal region of AnkA potentially serves as an NLS or possesses NLS-like function [147]. As a result, it was concluded that the N-terminal region of AnkA is necessary for the translocation of AnkA to the nucleus [147]. After AnkA translocates to infected cell nuclei, it binds to host DNA in a sequence-independent manner at regions of long stretches of A, T, and C nucleotides. As a result of AnkA binding to these matrix attachment regions (MARs), AnkA induces 3-dimensional alterations in chromatin organization that modulates host transcriptional events, such as transcription of genes involved in granulocyte respiratory burst [148]. AnkA is described to specifically target and bind regions of the *CYBB* promotor that are similar to MARs in both sequence and function [148]. By targeting the *CYBB* promotor, AnkA initiates recruitment of histone deacetylase-1 (HDAC1), leading to downstream deacetylation of histone H3 [147]. Unlike LntA of *L*. *monocytogenes*, AnkA acts by recruiting HDAC-associated complexes as opposed to inhibiting them [3]. By HDAC1-mediated deacetylation of histone H3, AnkA indirectly alters binding interactions with RNA polymerase 2 and results in targeted silencing of genes and repression of granulocyte response to infection [147,148]. By altering chromatin organization and reprogramming the transcriptional landscape of host cells with AnkA interactions, *A*. *phagocytophilum* creates an environmental niche favorable for prolonged intracellular survival.

Like *A*. *phagocytophilum*, *Ehrlichia* spp. (*E*. *chaffeensis* and *E*. *canis*) are Rickettsial pathogens that secrete a nucleomodulin with similar function as AnkA, known as Ank200 (or p200) [3,150]. However, Ank200 differs from AnkA due to being a T1SS effector protein as opposed to a T4SS effector protein [3,151]. Ank200 was found to translocate to the nuclei of mononuclear host cells, such as monocytes and macrophages, but lacks a classical NLS when analyzed with prediction software [150,152]. While Ank200 lacks a known DNA binding domain, Ank200 is suggested to directly associate with host chromatin or indirectly associate with chromatin via protein–protein interactions [150]. Once inside the host cell nucleus, Ank200 binds and interacts with *Alu-sx* element motifs of promotor and intron segments of host genes involved in ATPase activity, apoptosis, gene transcription, gene translation, cell response and signaling, cytoskeletal rearrangement, structural proteins of organelles, and intracellular trafficking [3,129,150,152]. *Alu-sx* element motifs are nonrandom, repetitive AT-rich DNA regions comprising nearly 10% of the human genome but are more commonly found within the 5-kb upstream region of gene transcription start sites [3,150,152]. Because genes associated with host cell processes and immune response are down-regulated during *Ehrlichia* infection, it is hypothesized that Ank200 induces large-scale transcriptional alterations through direct association with *Alu-sx* element motifs of host DNA [3,150,152]. By secreting Ank200, *Ehrlichia* spp. induces epigenetic modulation within the host cell, consequently inhibiting the cellular immune response and promoting pathogenesis. A more comprehensive understanding of Ank200’s association with host chromatin and *Alu-sx* element motifs is necessary to determine if Ank200 provides *Ehrlichia* spp. with multiple mechanisms for modulating host cell gene expression.

Tandem repeat protein (TRP) 32, TRP47, and TRP120 are type one (T1)-secreted effectors from *E*. *chaffeensis* that share similar activity as Ank200 but reportedly target and bind to specific G- or GC-rich DNA motifs [3,151–153]. The mechanism used by TRP32 and TRP120 to localize to host cell nuclei has yet to be described, but it is noted that TRP32 nuclear localization is dependent on TRP32 phosphorylation [154]. Within the nucleus, TRP32 and TRP120 act as multitargeted effectors capable of recognizing and binding host DNA, chromatin-associated proteins, histone methylases and demethylases, polycomb-group (PcG) proteins, and other substrates that are involved in chromatin remodeling complexes [155]. However, the primary target supporting their nucleomodulin activity is DNA. TRP32 specifically targets and binds G-rich motifs on DNA and either increases or represses the expression of genes related to immune cell differentiation, chromatin remodeling, and RNA transcription events [129,155]. TRP120 targets and binds GC-rich DNA motifs and serves as a transcriptional activator of host genes associated with transcriptional regulation, signal transduction, and apoptosis [3,129]. While the binding targets of TRP32 and TRP120 have been identified, their specific mechanism of action and how they alter host gene expression is still unknown. TRP47 is the fourth nucleomodulin discovered in *E*. *chaffeensis*. Studies in HeLa cells have determined nuclear localization of TRP47 to be dependent on a MYND (Myeloid, Nervy, DEAF-1)-binding domain (MBD) and potential interactions with NLS-containing host proteins [3,154]. Because the MBD is also a zinc finger motif, it is hypothesized that TRP47 uses its MBD for protein–protein interactions with host transcription regulatory proteins [154]. Once inside the nucleus, TRP47 is suggested to closely resemble TRP120 DNA binding activity and target genes involved in vesicular trafficking, signal transduction, and host immune response [154]. TRP47 reportedly shares similar characteristics as TRP32, TRP120, and the TAL family of effectors [154]. Together, TRP32, TRP47, and TRP120 illustrate how individual pathogens can secrete closely related proteins with different target sites and function to facilitate changes to the host cell epigenome. By utilizing TRPs, *E*. *chaffeensis* modulates the expression of host genes and establishes an intracellular environment to promote pathogenesis. Further studies will be necessary to characterize the extent of TRP32, TRP47, and TRP120 epigenetic modulation of host cells.

Another pathogen that secretes a large repertoire of proposed nucleomodulins with predicted NLSs (classical or unclassical) is *Coxiella burnetii* [156–158]. Effector proteins secreted by *C*. *burnetii* through a T4SS, and identified to translocate to host cell nuclei, are: Cbu0129, Cbu0388, Cbu0393, Cbu0781 (AnkG), Cbu0794, Cbu1314, Cbu1524 (CaeA), and CbuK1976 [156–164]. While these potential nucleomodulins are identified to localize within the nucleus during in vitro studies and contain predicted NLSs (classical and unclassical), only three of these nucleomodulins (Cbu1314, CaeA, and AnkG) are further characterized and have their nuclear substrate targets identified. However, while CaeA and AnkG have been linked to intranuclear interactions that delay host cell apoptosis and targeting of nuclear proteins for alteration of cell processes (gene translation, gene splicing, RNA transport, RNA transcription, and ubiquitin-proteasome regulation), how these effectors modulate nuclear function in vivo has yet to be addressed [158,159]. So far, in vitro studies with macrophages and HEK293 cells reveals that interactions with host protein p32 and Importin-α1 facilitate the nuclear import of AnkG, while CaeA relies on 2 different NLSs [158,165]. On the other hand, Cbu1314 is a nucleomodulin conserved among *C*. *burnetii* pathotypes and was recently discovered to directly associate with chromatin and modulate the host transcriptome [129,157,163]. Cbu1314 possesses 6 potential NLSs with the 52 to 75 residues and 181 to 186 residues necessary for nuclear import [157]. While the specific molecular mechanism of action performed by Cbu1314 has yet to be explored, studies have identified that Cbu1314 shares motifs used by *A*. *phagocytophilum* (AnkA) and *Ehrlichia* spp. (Ank200/p200 and tandem-repeat containing protein 120 (TRP120)) within identified target sequences for binding with AT-rich DNA regions, *Alu-sx* elements, and GC-rich regions, respectively [157]. Studies transfecting HEK293 and HeLa cells with Cbu1314 suggest that Cbu1314 modulates the host transcriptome by inducing expression of antiapoptotic genes via chromatin complexes and directly associating with genes that encode zinc finger proteins, microRNAs, ubiquitination machinery, immune response, and intracellular transport and vesicular trafficking [157]. How Cbu1314 specifically manipulates host gene expression in vivo has yet to be fully explored. However, the large repertoire of nucleomodulins secreted by *C*. *burnetii* provides *C*. *burnetii* with multiple strategies for manipulation of the host transcriptome and maintenance of a novel intracellular niche for pathogen proliferation.

### Methylation of host DNA by nucleomodulins

While the previously described nucleomodulins for *A*. *phagocytophilum*, *Ehrlichia* spp., and *C burnetii* induce epigenetic events in host cells by directly binding to DNA, other nucleomodulins have evolved to target DNA and modulate the host epigenome by serving as mammalian DNA methyltransferases (DNMTs). These unique DNMT nucleomodulins are identified as effector proteins secreted by *M*. *hyorhinis* and *M*. *tuberculosis* [3]. *M*. *hyorhinis* secretes 3 DNMTs known as Mhy1, Mhy2, and Mhy3 [3,66,166,167]. While all three have been found to translocate to host cell nuclei, studies have yet to determine if Mhy1, Mhy2, and Mhy3 carry classical or unclassical NLSs [166,167]. After localization into host cell nuclei, these *M*. *hyorhinis* DNMTs target specific recognition sites of host DNA and generate methylated sites that serve as epigenetic modifications [167]. Infection of HTR8/SV neo trophoblasts identified CG-rich sites of human DNA as targets for Mhy1 and Mhy2 methylation, while GATC-rich sites were targets for Mhy3 methylation [3,129,166,167]. While the methyltransferase activity of Mhy1, Mhy2, and Mhy3 in vivo has yet to be fully addressed, these DNMTs influence an up- and down-regulation of host genes that regulate proliferation-specific pathways [66,166]. Because *M*. *hyorhinis* is found during colorectal cancer, these nucleomodulins are hypothesized to play a role in methylating cancer-associated genes and promoting tumor progression [3,66,166,167]. By altering host DNA through DNMTs and inducing long-term epigenetic modifications, *M*. *hyorhinis* may create an environmental niche favoring proliferation and facilitating further cell-to-cell dissemination.

Like the above *M*. *hyorhinis* nucleomodulins, Rv2966c secreted by *M*. *tuberculosis* functions as a DNMT. Because Rv2966c lacks an identifiable NLS, it hypothesized that residues on the C-terminus of Rv2966c interact with host cell proteins and regulate its trafficking into host cell nuclei [168]. Studies in HEK293 cells suggested Rv2966c utilizes host cell NPM1 for nuclear localization due to its function as a nucleo-cytoplasmic shuttling protein [168]. Once in the nucleus, Rv2966c reportedly targets and methylates regions of DNA at cytosine residues in a non-CpG manner [3,168]. By serving as a novel mechanism to alter host DNA, Rv2966c modulates the host epigenome and represses transcription of host genes [3,168]. Rv2966c has also been identified to target and modify histones H3 and H4, but its mechanism of histone modification has yet to be determined [168]. Other than the nucleomodulins found in *M*. *hyorhinis* and *M*. *tuberculosis*, there has yet to be an identification of other bacterial DNMTs that target host cell DNA [3].

### Modification of histones by nucleomodulins

Bacterial pathogens utilize nucleomodulins to directly target and alter histones in eukaryotic host cells. Pathogens identified to modify histones through nucleomodulins are: *Chlamydia* spp. (*C*. *trachomatis* and *C*. *pneumoniae*), *L*. *pneumophila*, *Burkholderia* spp. (*B*. *thailandensis* and *B*. *pseudomallei*), *Bacillus anthracis*, *M*. *tuberculosis*, and *Neisseria meningitidis*. NUE is a SET domain-containing nucleomodulin secreted by *C*. *trachomatis* through a T3SS and is identified to be the first bacterial effector that mimics host histone methyltransferases [1,3,169]. NUE is also one of only 2 SET domain-containing effectors capable of automethylation to enhance methyltransferase activity by potentially increasing NUE’s affinity to host target substrates [169]. Both NUE and its homologue (cpnSET from *C*. *pneumoniae*) reportedly contains an NLS for localization within host cell nuclei [1,3,14,169]. Once in the nucleus, NUE functions as a histone lysine methyltransferase (HKMTase) to target and methylate host histones H2B, H3, and H4 [1,3,66,169]. As a result of histone methylation observed in HeLa cell studies, NUE is suggested to alter host chromatin structure and gene regulation [3,169]. However, specific host genes altered by NUE histone methylation have yet to be identified. The cpnSET homologue of NUE was found to target and methylate histone-like proteins Hc1 and Hc2 along with mouse histones during murine studies [14]. Even though the molecular mechanisms utilized by NUE to influence the host epigenome have yet to be fully characterized in vivo, the identification of NUE in *C*. *trachomatis* is considered a pioneering discovery in the studies of SET domain-containing effectors [3].

After the discovery of NUE in *C*. *trachomatis*, 2 SET domain-containing homologue effectors known as Regulator of methylation A (RomA, from *L*. *pneumophila Paris* strain) and LegAS4 (from *L*. *pneumophila Philadelphia* strain) were described. Like NUE, RomA/LegAS4 exhibit HKMTase activity towards host cell histones [14,140,141]. RomA was the first T4SS-secreted nucleomodulin identified in *Legionella* and the first bacterial effector described to induce new epigenetic marks on the chromatin landscape of host cells [17,170]. Like many nucleomodulins, RomA contains an NLS in the N-terminal region of its sequence to promote nuclear localization [1,170,171]. Upon translocation to the host cell nucleus, RomA specifically targets and tri-methylates the Lys14 residue of histone H3 (H3K14) [14,17]. It is important to note that while acetylation and deacetylation regulation of histone H3K14 has been noted in mammalian cells, the methylation of histone H3K14 was never previously described [1,14,17]. This suggests that RomA of *Legionella* has evolved a novel mechanism to induce a novel epigenetic modification and subsequent inhibition of select gene transcription in host cells [1,14,17]. It was also discovered that this novel epigenetic change induced by RomA occurs within the amoeba hosts of *L*. *pneumophila*, indicating a coevolutionary targeting of a highly conserved eukaryotic process [170]. By methylating histone H3K14, RomA inhibits global transcription and negatively regulates the innate immune response of the host cell [1,3,66]. Like RomA, LegAS4 possesses an N-terminal NLS. However, the NLS of LegAS4 differs from its RomA homologue by containing 13 extra amino acids on the N-terminus [171]. While LegAS4 shares histone H3K14 methylation activity with RomA, LegAS4 differs from RomA by targeting and methylating histone H3K4 [3,14,17]. LegAS4 is also hypothesized to interact with HP1 in the nucleolus at rDNA promotors, resulting in an activation of rDNA gene expression [3,172,173]. By utilizing RomA or LegAS4, *L*. *pneumophila* strains have evolved to induce epigenetic changes within the host cell transcriptional landscape, repressing the host immune response and promoting pathogenesis.

The identification of LegAS4 and its function led to the later description of a LegAS4-like nucleomodulin with similar function. BtSET is a LegAS4-like nucleomodulin secreted by pathogenic *B*. *pseudomallei* and nonpathogenic *B*. *thailandensis* through one of their T3SSs known as the *Burkholderia* secretion apparatus (Bsa) [3,174,175]. While BtSET has been confirmed to localize to the nucleolus of infected cells, a definitive NLS has yet to be identified [140]. Like LegAS4, BtSET has H3K4 methylation activity in HeLa cells and perform mono-/di-methylation of rDNA [1,140]. By methylating rDNA, BtSET activates transcription of rDNA genes [1,140]. The shared function of H3K4 methylation in the nucleolus between *Burkholderia* and *Legionella* nucleomodulins highlights the potential evolution of a shared virulence strategy among different bacterial pathogens promoting dysregulation of host ribosomal machinery and establishing an environmental niche favorable for pathogen replication and pathogenesis.

Along with methylation of H3K4 and H3K14, H1 methylation is another unusual epigenetic modification that has yet to be identified in mammalian cells under normal circumstances. The nucleomodulin responsible for this modification, BaSET secreted by *B*. *anthracis*, localizes to the nuclei of infected HeLa cells, HEK293 cells, and macrophages [14,176–178]. The mechanism for nuclear translocation of BaSET has yet to be identified. After reaching the nucleus, BaSET functions as a specific histone tri-methylase via targeting of 8 lysine residues of histone H1 [1,14,178]. Research assessing BaSET-mediated histone H1 methylation led to the hypothesis that the activity of BaSET in host cell nuclei results in transcriptional repression of inflammatory genes [176]. This hypothesis is supported by the identification of repressed NF-κB target gene promotors after overexpression of BaSET in mammalian cells [66,176,177]. Without the secretion of BaSET and subsequent repression of the host inflammatory response, *B*. *anthracis* is unable to survive within host cells [176,177]. This suggests that BaSET has evolved as an essential nucleomodulin for promoting pathogenesis of *B*. *anthracis*.

Another nucleomodulin described to target and methylate host histones is Rv1988 of *M*. *tuberculosis*. Rv1988 is a second nucleomodulin secreted into infected host cells by *M*. *tuberculosis* [3,66,179,180–183]. Because of a Tat-signal sequence present within the N-termini of Rv1988, it is suggested that Rv1988’s secretion is dependent on the Tat secretion pathway of *M*. *tuberculosis* [179]. As for Rv1988’s localization within the nucleus, studies show the nuclear localization of Rv1988 in macrophages is dependent on 3 sections of basic amino acids located within its C-terminal sequence [179]. Once within the nucleus, Rv1988 acts as a unique histone methyltransferase that targets and di-methylates a noncanonical arginine residue (R42) in histone H3 (H3R42) [3,66,179,180]. It is important to note that histone H3R42 is located at a crucial entry/exit region of DNA in the host nucleosome [179]. As a result of this location, H3R42 has the potential to induce dynamic changes within the nucleosome structure and alter cellular transcription events [179]. Rv1988’s secretion from *M*. *tuberculosis* and its interaction with host epigenetic machinery is hypothesized to modulate multiple genes responsible for host immune response [179,180,184–186]. This hypothesis is supported by a decrease in ROS activity of THP1 macrophages and decreased expression of NADPH oxidase (*NOX1* and *NOX4*) and nitric oxide synthase (*NOS2*)) genes correlating with Rv1988 methylation of H3R42 [3,179]. Rv1988 methylation of H3R42 also represses *TRAF3*, a TNF receptor-associated factor which plays a crucial role in host immune response mediated by B cells and T cells [179]. However, the influence Rv1988 has over B cell and T cell immune response has yet to be as thoroughly studied as in macrophages. By targeting regulatory elements of the host cell genome and modulating host transcription, Rv1988 serves as an important virulence factor for promoting pathogenesis and persistence of *M*. *tuberculosis* [187].

*M*. *tuberculosis* secretes a third nucleomodulin identified as Rv3423, but unlike Rv2966 and Rv1988 it functions as a novel histone acetyltransferase (HAT) in vitro as opposed to a DNA or histone methyltransferase, respectively [3,180,188]. Rv3423 is described to only be secreted by the virulent strain of *M*. *tuberculosis* [188]. This is hypothesized to occur as a result of an ESX-1 type VII secretion system (T7SS) present and unimpaired in the virulent strain of *M*. *tuberculosis* as opposed to the avirulent strain [188,189]. While Rv3423 localizes within the nuclei of *M*. *tuberculosis*-infected macrophages, a predicted NLS has yet to be identified. Due to Rv3423’s small size, Rv3423 is hypothesized to traffic to the nucleus via diffusion through nuclear pores or hijack host nuclear trafficking proteins for transport [188]. Within the nucleus, Rv3423 targets and acetylates histone H3 at the lysine 9 (H3K9) and/or lysine 14 (H3K14) positions [3,180,188]. Since histone acetylation is involved in the regulation of gene transcription, Rv3423 is hypothesized to regulate host gene transcription in a way that promotes the intracellular survival of *M*. *tuberculosis* [14,17,188]. However, the specific role of Rv3423 during *M*. *tuberculosis* infection of macrophages has yet to be fully characterized. Altogether, the ability of *M*. *tuberculosis* to secrete Rv2966, Rv1988, and Rv3423 illustrates how bacterial pathogens have evolved multiple effector-mediated strategies that consequentially target and inhibit host response through various epigenetic modifications.

The above nucleomodulins have been described to target and modify mammalian histones by acting as histone methyltransferases and acetyltransferases. While this is the most commonly reported method for modification of host cell histones, some nucleomodulins modify histones by acting as proteases. *N*. *meningitidis* harbors 2 chemotrypsin-like serine proteases, adhesion and penetration protein (App) and meningococcal serine protease A (MspA/AusI), described to share homology with human IgA1 protease and be internalized by human dendritic cells (DCs) in vitro [190–193]. As type Va autotransporters, App and MspA are released from the surface of *Neisseria* via autoproteolytic cleavage, internalized by DCs through a mannose receptor-/transferrin receptor-mediated manner, and then translocated to the host cell nuclei [3,190,192]. The α-peptide of App contains 2 NLSs, like those found in host IgA1 protease, that are necessary for nuclear localization [190]. While MspA lacks an identifiable NLS, MspA still localizes within host cell nuclei [190]. How MspA localizes within cell nuclei without a predicted NLS has yet to be determined. Once in DC nuclei, App and MspA reportedly target and proteolytically cleave histone H3 in vitro, suggesting these nucleomodulins induce DC apoptosis in vivo [3,190,192,193]. While more research is necessary to bridge the connection between histone H3 cleavage and DC apoptosis, App and MspA are hypothesized to interfere with host pathways and promote *N*. *meningitidis* pathogenesis.

## Conclusions

Throughout the last 10 years, it has become increasingly evident that bacterial pathogens have evolved a remarkable and powerful mechanism for modulating host cell transcriptional regulation and gene expression. These unique nucleomodulins promoting pathogenesis were initially discovered in phytopathogens and have since been identified in mammalian pathogens. Here, we discussed an expansive number of injected or secreted nucleomodulins that mimic or directly target host transcriptional and transformation factors, ubiquitination machinery for regulating nuclear protein homeostasis, nuclear proteins for regulation of downstream signaling pathways, and host cell DNA and histones for modulating chromatin structure and gene transcription (Fig 1). While several of the described nucleomodulins harbor an NLS for nuclear trafficking, most nucleomodulins traffic to the nucleus via alternative mechanisms (Fig 1). Further studies are needed to uncover the novel mechanisms behind the nuclear trafficking of nucleomodulins lacking an NLS.

It is important to note that while several of the described nucleomodulins share similar function, most known nucleomodulins are idiosyncratic in target substrate and pathway specificity. Some of the first identified nucleomodulins (VirD2, TALENs, HsvG) function by mimicking eukaryotic host transcription and transformation factors to regulate host transcription activation [2,34]. Nucleomodulins such as Ank1 from *O*. *tsutsugamushi* or IpaH_9.8_ from *S*. *flexneri* function by mimicking or directly inhibiting components of intranuclear ubiquitination pathways to alter nuclear protein homeostasis [68]. LntA of *Shigella* and SinC of *Chlamydia* are 2 examples of novel functioning nucleomodulins that target different nuclear proteins for indirect influence of chromatin structure and regulatory processes, whereas AnkA of *Anaplasma* binds directly to specific regions of DNA [123,134,150]. Finally, there are nucleomodulins such as LegAS4 of *Legionella* and BtSET of *Burkholderia* which are examples of proteins utilized by different bacterial species that share close function by methylating histone H3K4 [140].

The mimicry of eukaryotic host factors, along with the shared targeting and function observed between several nucleomodulins, suggests convergent evolution between both eukaryotic cells and bacterial pathogens. While it is understandable to speculate the functional redundancy of nucleomodulins is the result of natural selection or interkingdom horizontal gene transfer, there is still the question regarding how nucleomodulin mimicry of eukaryotic factors came to be [14,170]. Nucleomodulins illustrate a unique mimicry of eukaryotic factors when they possess an NLS, Ank, SET, or LRR domain and share the same binding partners as eukaryotic nuclear proteins. Could pathogens have acquired homologues of eukaryotic proteins through interkingdom horizontal gene transfer that have evolved into unique nucleomodulins capable of mimicking eukaryotic factors that we find today? It is still unknown how nucleomodulins first came to be, and further studies are needed to comprehend why some pathogen nucleomodulins mimic eukaryotic factors (NLS, Ank, SET, etc.) while others are novel in their identity and function.

Nucleomodulin secretion is an emerging theme shown to promote pathogen-induced epigenetic modification and control of the host cell epigenome via epigenetors. While the list of discovered nucleomodulins is rapidly expanding (Tables 1 and 2), there is no doubt that the current number of identified nucleomodulins is merely the tip of the iceberg among bacterial pathogens. On top of this, the mechanisms used by many nucleomodulins for nuclear trafficking in the absence of an NLS and modulation of the host genome are still unknown. The ability of nucleomodulins to mimic eukaryotic proteins along with related functions of nucleomodulins between different pathogens suggests an evolutionary convergence that has yet to be thoroughly explored. A further understanding of this unique strategy evolved in pathogens for promoting pathogenesis will lay groundwork for an expansive field of research regarding nucleomodulin function and host cell impact. Uncovering these mysteries on unique pathogen weaponry will result in an enhanced understanding of how nucleomodulins came to be a rising enigma of host–pathogen interactions.

**Table 2:** Mammalian pathogen effectors that target the nucleus.

Pathogen	Effector	Effector Function	Source
*Anaplasma*	AnkA	Binds to long stretches of A, T, and C nucleotides of chromatin; induces change in chromatin structure, promotes deacetylation of histone H3, and silences genes for host cell response	[147,148]
*Bacillus*	BaSET	Histone tri-methylase; targets 8 lysine residues of histone H1 for transcriptional repression	[176–178]
*Bordetella pertussis*	BopN	Suggested to bind or promote export of p65 from nucleus to manipulate the host NF-κB pathway; promotes nuclear translocation of p50	[68,69]
*Burkholderia*	BtSET	Histone lysine methyltransferase; targets H3K4 and nucleolar rDNA	[1,140]
*Chlamydia psittaci*	SinC	Interacts with ELYS, laminB1, LEM domain proteins, LAM1, and LBR on the inner nuclear membrane; indirectly modulates chromatin structure and silences inflammatory genes	[133–135]
*Chlamydia*	cpnSET	Histone lysine methyltransferase; targets histone-like proteins Hc1 and Hc2	[14]
	NUE	Histone lysine methyltransferase capable of automethylation; targets histones H2B, H3, and H4	[66,169]
*Coxiella burnetii*	Cbu0129	Unknown	[156]
	Cbu0388	Unknown	[156,163]
	Cbu0393	Unknown	[156]
	Cbu0781 (AnkG)	Target nuclear proteins to alter host cell processes and delay apoptosis	[158,159]
	Cbu0794	Unknown	[156,163]
	Cbu1314	Binds chromatin at target sequences with AT-rich regions, *Alu-sx* elements, and GC-rich regions; induces expression of antiapoptotic genes	[157,194]
	Cbu1524 (CaeA)	Target nuclear proteins to alter host cell processes and delay apoptosis	[158,159]
	CbuK1976	Unknown	[194]
*Ehrlichia chaffeensis*	Ank/p200	Associates with chromatin by targeting AT-rich *Alu-sx* motifs of host genes; induces large-scale transcriptional alterations	[150,152]
	TRP32	Binds to G-rich DNA motifs	[129,155]
	TRP47	Binds to GC-rich DNA motifs	[154]
	TRP120	Binds GC-rich DNA motifs; acts as a transcriptional activator	[3,129]
*Escherichia coli*	Cif	Cyclomodulin; exhibits deaminase activity towards NEDD8 and inhibits CRL activity necessary for cell cycle progression	[97,98]
	EspF	Targets the nucleolus; induces redistribution of nucleolin and inhibits ribosome biogenesis	[99,102]
	NleC	Zinc metalloprotease; cleaves p300, RelA, RelB, cRel, NF-κB1, and NF-κB2 nuclear transcription factors to modulate the NF-κB signaling pathway and regulate host transcription	[18,121]
	NleG5-1	U-box E3 ubiquitin ligase activity; targets MED15 and disrupts transcription signaling pathways	[90,91]
*Legionella*	AnkH	Controls RNA polymerase II activity; interacts with LARP7 and inhibits host cell transcription	[140,141]
	AnkX	Interacts with PLEKHN1; manipulates host inflammatory response	[146]
	LegAS4	Histone lysine methyltransferase; targets histones H3K14 and H3K4 to inhibit global transcription. Suggested to interact with HP1 at rDNA promotors in the nucleolus	[14,17]
	RomA	Histone lysine methyltransferase; targets histone H3K14 to inhibit global transcription.	[14,17]
	SnpL	Controls RNA polymerase II activity; binds SUPT5H and upregulates gene expression for fundamental biological processes	[139]
*Listeria monocytogenes*	LntA	Targets and binds to the proline rich region of BAHD1 protein; inhibits BAHD1-mediated gene silencing and histone H3 acetylation	[23,123]
	OrfX	Targets host cell RYBP; results in inhibition of oxidative activity of macrophages	[66,124]
*Mycobacterium*	Rv1988	Histone methyltransferase; targets histone H3R42 and reduces expression of oxidative genes	[179,180]
	Rv3423	Histone acetyltransferase; targets histone H3K9 and H3K14	[180,188]
	Rv2966c	DNA methyltransferase activity; targets cytosine residues of DNA motifs in a non-CpG manner	[3,168]
*Mycoplasma*	Mhy1	DNA methyltransferase activity; targets GC-rich DNA motifs	[166,167]
	Mhy2	DNA methyltransferase activity; targets GC-rich DNA motifs	[166,167]
	Mhy3	DNA methyltransferase activity; targets GATC-rich DNA motifs	[166,167]
*Neisseria*	App	Chemotrypsin-like serine protease; targets and cleaves histone H3	[190–193]
	MspA	Chemotrypsin-like serine protease; targets and cleaves histone H3	[190–193]
*Orientia*	Ank1	Interacts with CULLIN-1 and SKP1 components of the SCF E3 ubiquitin ligase complex; suggested to bind or promote export of p65 from nucleus to manipulate the host NF-κB signaling pathway	[62,66–68]
	Ank6	Suggested to bind or promote export of p65 from nucleus to manipulate the host NF-κB signaling pathway	[68]
*Salmonella enterica*	GogA GtgA PipA	Zinc metalloproteases, cleave RelA, RelB, cRel nuclear transcription factors to modulate the NF-κB signaling pathway and regulate host transcription	[118,119]
	Ssph1	Contains an LPX and NEL domain; interacts with host cell PKN1 to inhibit the NF-κB signaling pathway	[85–87]
*Shigella flexneri*	IpaB	Cyclomodulin; interacts with Mad2L2 and inhibits cell proliferation	[75,77,78]
	IpaH_9.8_	Contains an LPX and NEL domain; ubiquitinates host U2AF mRNA splicing factor and represses proinflammatory genes	[70,72,73]
	OspB	Acts in synergy with OspF to target pRB and down-regulate host immune response	[66,71,130]
	OspC1	Modulates epithelial cell signaling for PMN migration	[125,126]
	OspF	Exhibits phosphothreonine lyase activity towards host cell MAPKs; prevents transcription activation of NF-κB-regulated genes. Acts in synergy with OspB to target pRB and down-regulate host immune response	[66,71,126,127,130]
*Yersinia*	YopM	Contains an LPX domain but lacks an NEL domain; interacts with and hyperphosphorylates RSK1 and PRK2 to inhibit pyrin inflammasome formation in cytosol, and co-opts RSK1 to increase expression of *IL-10* gene in the nucleus.	[105,107,108,113–116]

AnkA, ankyrin repeat protein A; App, adhesion and penetration protein; BAHD1, bromo adjacent homology domain-containing 1; Cif, cyclin-inhibiting factor; CRL, CULLIN-RING ubiquitin ligase; LAM1, lamin-associated polypeptide 1; LBR, lamin B receptor; LEM, LAP2, emerin, MAN1; LntA, Listeria nuclear targeted protein A; MAPK, mitogen-activated protein kinase; MspA, meningococcal serine protease A; PMN, polymorphonuclear neutrophil; pRB, retinoblastoma protein; RomA, Regulator of methylation A; RYBP, Ring1 YY1-binding protein; SCF, SKP1-CULLIN1-F-box; SinC, Secreted inner nuclear membrane-associated *Chlamydia*.
